# Hyperhomocysteinemia Promotes Cardiac Hypertrophy in Hypertension

**DOI:** 10.1155/2022/1486157

**Published:** 2022-08-22

**Authors:** Yawen Deng, Zhitong Li, Xiangbo An, Rui Fan, Yao Wang, Jiatian Li, Xiaolei Yang, Jiawei Liao, Yunlong Xia

**Affiliations:** ^1^Institute of Cardiovascular Diseases, First Affiliated Hospital of Dalian Medical University, Dalian, China; ^2^Department of Interventional Therapy, First Affiliated Hospital of Dalian Medical University, Dalian, China

## Abstract

Hyperhomocysteinemia (HHcy) is positively linked with several cardiovascular diseases; however, its role and underlying mechanisms in pathological cardiac hypertrophy are still unclear. Here, we focused on the effects and underlying mechanisms of HHcy in hypertensive cardiac hypertrophy, one of the most common and typical types of pathological cardiac hypertrophy. By a retrospective analysis of the association between HHcy and cardiac hypertrophy in a hypertensive cohort, we found that the prevalence of HHcy was higher in patients with hypertrophy and significantly associated with the presence of cardiac hypertrophy after adjusting for other conventional risk factors. In mice, HHcy induced by a methionine (2% wt/wt) diet feeding significantly promoted cardiac hypertrophy as well as cardiac inflammation and fibrosis induced by 3-week angiotensin ІІ (AngІІ) infusion (1000 ng/kg/min), while folic acid (0.006% wt/wt) supplement corrected HHcy and attenuated AngII-stimulated cardiac phenotypes. Mechanistic studies further showed that homocysteine (Hcy) exacerbated AngII-stimulated expression of Calcineurin and nuclear factor of activated T cells (NFAT), which could be attenuated by folic acid both in mice and in neonatal rat cardiomyocytes. Moreover, treatment with cyclosporin A, an inhibitor of Calcineurin, blocked Hcy-stimulated Calcineurin-NFAT signaling and hypertrophy in neonatal rat cardiomyocytes. In conclusion, our study indicates that HHcy promotes cardiac hypertrophy in hypertension, and Calcineurin-NFAT pathway might be involved in the pro-hypertrophic effect of Hcy.

## 1. Introduction

The main function of the heart is to pump blood to support the peripheral organs. In stress conditions, such as increased preload or afterload, the heart undergoes enlargement to increase its contractility and pumping capability, a process termed cardiac hypertrophy [1]. As a stress-induced adaptive change to maintain cardiac efficiency, cardiac hypertrophy could be classified into physiological and pathological hypertrophy, according to different types of stimuli. Unlike physiological hypertrophy, which is typically caused by pregnancy or exercise, pathological hypertrophy is however known to be induced by disease conditions, including inherited hypertrophic cardiomyopathy and other secondary triggers, such as pressure overload caused by hypertension or aortic stenosis, volume overload owing to chronic kidney diseases and mitral and aortic regurgitation, long-term myocardial hypoxia due to anemia, myocardial infarction, and chronic obstructive pulmonary disease [1]. More importantly, prolonged and excessive pathological hypertrophy is irreversible and eventually progresses to severe cardiovascular consequences, such as heart failure, arrhythmia, and death [1]. Therefore, unveiling the risk factors and underlying mechanisms of pathological cardiac hypertrophy is important for improving cardiovascular health and controlling adverse cardiovascular events.

Homocysteine (Hcy) is a sulfur-containing amino acid derived from methionine (Met) metabolism [2, 3]. As an essential amino acid, Met is released from dietary protein, absorbed by the digestive tract epithelium, and transported in the circulation to various organs, where it is metabolized into cysteine through remethylation and transsulfuration pathways via several key enzymes, such as cystathionine *β*-synthase, methionine synthase, or methylenetetrahydrofolate reductase [2, 3]. Genetic defects impeding the activity of these enzymes disrupt Met metabolism and cause elevation of circulating Hcy [2, 3]. Moreover, diets rich in Met or lack of vitamin B6, B12, or folate, cofactors for the abovementioned enzymes, also impair Met homeostasis, leading to systemic accumulation of Hcy [2, 3]. Mounting evidence has suggested that hyperhomocysteinemia (HHcy, defined as serum/plasma Hcy concentration ≥ 15 *μ*mol/L) due to either genetic or nutritional causes is detrimental for cardiovascular health, facilitating the onset and development of various vascular diseases, such as hypertension, atherosclerosis, and aneurysm, and therefore is identified as an independent metabolic risk factor for these diseases [4, 5]. However, whether and how HHcy contributes to pathological cardiac hypertrophy is still unclear.

Here in this study, we focus on the effects and underlying mechanisms of HHcy in hypertensive cardiac hypertrophy, one of the most common and typical types of pathological cardiac hypertrophy. We first analyzed the correlation between HHcy and hypertensive cardiac hypertrophy in a cohort study enrolling 2101 hypertensive patients. We then directly explored the causative role of HHcy as well as the therapeutic potent of folate acid (FA) therapy on experimental hypertensive cardiac hypertrophy induced by angiotensin ІІ (AngІІ) both *in vivo* and *in vitro*. Our data suggested that HHcy might be a potential risk factor for this type of pathological cardiac hypertrophy.

## 2. Methods and Materials

### 2.1. Retrospective Cohort Study

#### 2.1.1. Study Participants

We retrospectively enrolled hypertensive patients hospitalized in the First Affiliated Hospital of Dalian Medical University between August 1, 2015, and June 30, 2021. Patients with organic valvular heart disease, rheumatic heart disease, prosthetic valve placement, or malignant tumor were excluded from analysis. Moreover, those with missing or incomplete echocardiography as well as laboratory data were also excluded. Finally, 2101 patients were eligible and divided into cardiac hypertrophy and nonhypertrophy cohorts for further analysis. The design and procedures of the retrospective study were approved by the institutional review board of Dalian Medical University (ChiCTR-1900021163), and the requirement for informed consent was waived. The research was conducted in accordance with the Helsinki Declaration guidelines, and all procedures listed here were performed in compliance with the approved guidelines.

#### 2.1.2. Data Source and Definition of the Explanatory Variables

Demographics, medical history, and laboratory data of the study participants were all collected from the electronic medical record of the hospital. Cardiac hypertrophy, referred to as left ventricular hypertrophy (LVH) in particular, was visualized by transthoracic echocardiography with a Vivid 7 ultrasound system (GE Healthcare, Horten, Norway) and defined as interventricular septal thickness (IVST)/left ventricular posterior wall thickness (LVPWT) > 10.5 mm in women or >11 mm in men [6]. Left ventricular mass index, an increasingly accepted criterion to diagnose LVH, is however not used in the current retrospective analysis, as the calculation of this index could not be done without the height data of the participants, which are not recorded routinely in the hospital. All echocardiographic measurements were performed and interpreted by experienced physicians in a blinded manner. Other explanatory variables include diabetes mellitus, defined as fasting glucose level ≥126 mg/dL, nonfasting glucose ≥ 200 mg/dL, a physician diagnosis of diabetes, or use of diabetes medication [7]; congestive heart failure (CHF), defined by a physician diagnosis of heart failure, or use of antiheart failure medication [8]; coronary artery disease (CAD), defined by a history of physician-diagnosed myocardial infarction, or coronary artery bypass surgery, or coronary angioplasty; HHcy, defined as circulating Hcy concentration ≥ 15 *μ*mol/L [9]; dyslipidemia, defined as circulating total cholesterol (TC) ≥ 6.22 mmol/L, low-density lipoprotein cholesterol (LDL − C) ≥ 4.14 mmol/L, high-density lipoprotein cholesterol (HDL − C) ≤ 1.04 mmol/L, triglycerides (TG) ≥ 2.26 mmol/L, or use of antidyslipidemia medication; moderate to severe left ventricular systolic dysfunction, defined as left ventricular ejection fraction (LVEF) ≤ 40%, according to the 2010 European Society of Cardiology guidelines [10]; left atrial enlargement (LAE), defined as left atrial diameter > 38 mm in women or >40 in men [11]; and renal insufficiency, defined as estimated glomerular filtration rate (eGFR) < 90 mL/(min•1.73 m^2^) [12].

#### 2.1.3. Statistical Analysis

Statistical analyses were performed using SPSS software, and a *p* value < 0.05 was considered as statistically significant. Categorical data were presented as count (percentage) and analyzed by *χ*^2^ test or Fisher exact test. Continuous variables were tested for normal distribution with Kolmogorov–Smirnov test. As all continuous variables presented in the study were nonnormally distributed, these variables were presented as medians (interquartile ranges) and analyzed by Mann–Whitney test. A logistic regression analysis was used to investigate the risk factors for cardiac hypertrophy in the enrolled hypertensive patients. The odds ratios (ORs) with 95% confidence intervals (CIs) were presented. Variables were first included in the univariate analysis, and those variables associated with cardiac hypertrophy at a significant level in the univariate analysis were candidates for multivariate analysis. For quartile analysis, patients were stratified into four quartiles based on serum Hcy levels. The respective cut-offs of serum Hcy levels for Q1, Q2, Q3, and Q4 were ≤11.67, 11.67-14.56, 14.56-18.61, and >18.61 *μ*mol/L, respectively. The likelihood of left ventricular hypertrophy (LVH) associated with serum Hcy levels was calculated using a logistic regression model. Hcy values were entered in the model as quartiles (with the Q1 quartile as the baseline reference) to assess the ORs and 95% CIs.

### 2.2. Experimental Study

#### 2.2.1. Materials

Hcy (H4628) was purchased from Sigma-Aldrich (St. Louis, MO, USA). Folic acid (FA) (S4605) and cyclosporin A (CsA) (S2286) were purchased from Selleckchem (Houston, TX, USA). AngII (A107852) was purchased from Aladdin (Shanghai, China). Antibodies against transforming growth factor-*β* (TGF-*β*) (3711S), Calcineurin (2614S), nuclear factor of activated T cells (NFAT) (2183S), and *α*-actinin (6487T) were all purchased from Cell Signaling Technology (Boston, MA, USA). Antibodies against phosphorylated and total nuclear transcription factor-*κ*B (NF-*κ*B) p65 were purchased from WanleiBio (WL02169; Shenyang, China) and Arigobio (ARG65677; Taiwan, China), respectively. Antibody against *β*-tubulin (10094-1-AP) was purchased from Proteintech (Wuhan, China).

#### 2.2.2. Animals

C57BL/6 mice (male, 4-5 weeks) were purchased from Beijing Vital River Laboratory (Beijing, China). Mice were housed in individually ventilated cages and fed with diet and water *ad libitum*. Methionine (Met) diet used in this study contains 2% wt/wt Met [13, 14], while FA-supplemented Met diet contains additional 0.006% wt/wt FA [15, 16]. Cardiac hypertrophy in mice was induced by subcutaneous AngII infusion at a dose of 1000 ng/kg/min using osmotic mini-pumps (Alzet Model 1007D; Durect Corp, Cupertino, CA, USA) for three weeks [17]. The housing, care, and experimental procedures were performed in accordance with the Guide for the Care and Use of Laboratory Animals published by the U.S. National Institutes of Health (NIH Publication No. 85-23, revised 1996) and approved by the Animal Care and Use Committee of Dalian Medical University (AEE20029).

#### 2.2.3. Blood Pressure and Echocardiography Analysis

Systolic blood pressure (SBP) was measured by a noninvasive tail-cuff system (BP-2010; Softron, Tokyo, Japan) and averaged from ten measurements per mouse [18]. Transthoracic echocardiography was performed using a high-resolution microultrasound system (Vevo 770; Visual Sonics, Toronto, Ontario, Canada) under anesthetization with 1.5% isoflurane inhalation [19]. Left ventricular (LV) ejection fraction (EF) and fractional shortening (FS) were determined using parasternal short axis M-mode imaging and averaged from three cardiac cycles [19].

#### 2.2.4. Histopathological Analysis

Mice were sacrificed by CO_2_ inhalation and flushed by phosphate buffer saline (PBS) through the left ventricle. The entire hearts were removed and weighted. After fixation in 4% paraformaldehyde solution (Life-iLab, Shanghai, China), hearts were embedded in paraffin and sectioned at five *μ*m thickness. Cardiac gross morphology was visualized by hematoxylin and eosin (H&E) staining (G1120; Solarbio, Beijing, China). Myocyte cross-sectional areas were visualized by rhodamine-labeled wheat germ agglutinin (WGA) (1.25 mg/mL; ZD0510, Vector Laboratory, Burlingame, CA, USA) staining and calculated by measuring over 200 cells collected over 10 random fields. Cardiac accumulation of inflammatory macrophages and fibroblasts were visualized by immunohistochemical staining (SP0041; Solarbio, Beijing, China) using antibodies against CD68 (diluted at 1 : 200; ab201340; Abcam, London, UK) and *α*-SMA (diluted at 1 : 200; 19245S; CST, Boston, MA, USA), respectively. Cardiac fibrosis was visualized by Masson's trichrome staining (G1340; Solarbio, Beijing, China). All quantifications were performed with ImageJ software.

#### 2.2.5. Cell Culture

Neonatal rat cardiomyocytes (NRCMs) were obtained from newborn Sprague-Dawley rats, as previously described [20]. In brief, newly extracted heart tissues were placed into a 100 mm culture dish loaded with adequate precooled PBS, cut into tiny pieces, and dissociated by 0.04% trypsin to single cell suspensions at 37°C. After plating in 5% CO_2_ and 37°C incubator for two hours, suspended cardiomyocytes were collected and transferred into 100 mm dishes and 24-well plates with coated laminin (10 *μ*g/mL). After cultured in DMEM/F12 supplemented with 10% fetal bovine serum for 48 hours and then serum-starved for at least twelve hours, plated cardiomyocytes were subjected to DMEM/F12 supplemented with Hcy (200 *μ*M) or Hcy (200 *μ*M) + FA (100 *μ*mol/L) or Hcy (200 *μ*M) + CsA (50 *μ*g/mL) or same amount PBS as control for the next twelve hours and then AngII (1 *μ*M) for 48 hours before harvested for *α*-actinin staining or protein extraction.

#### 2.2.6. Quantitative Real-Time PCR Analysis

Total RNA was extracted with Triquick Reagent (R1100; Solarbio, Beijing, China) and reverse-transcribed to complementary DNA using a RT kit (MedChemExpress, Monmouth Junction, NJ, USA) [21]. Quantitative real-time PCR was performed with SYBR Green qPCR reagents (MedChemExpress, Monmouth Junction, NJ, USA), using primers listed in Table S1. All samples were quantitated using the comparative CT method and normalized to *β*-actin.

#### 2.2.7. Western Blot Analysis

Proteins were extracted using RIPA buffer (R0020; Solarbio, Beijing, China). Total protein concentration of each extract was determined using a bicinchoninic acid protein assay kit (PC0020; Solarbio, Beijing, China). After normalization for total protein concentration, the protein lysates were separated by electrophoresis in SDS-PAGE gels (Life-iLab, Shanghai, China) and transferred to polyvinylidene difluoride membranes. The membranes were incubated with primary antibodies (diluted at 1 : 500) at 4°C overnight and then with horseradish peroxidase-conjugated secondary antibodies (1 : 3 000) for one hour at room temperature. All blots were developed by ECL assay (AP34L024; Life-iLab, Shanghai, China), and signal intensities were analyzed with ImageJ software.

#### 2.2.8. Statistical Analysis

Data were presented as mean ± standard deviation (SD). The Shapiro–Wilk test was used to determine whether data were normally distributed. Statistical comparisons were performed using two-way ANOVA, followed by Tukey's test for multiple comparisons. All statistical analyses were performed using GraphPad Prism software, and a *p* value < 0.05 was considered as statistically significant.

## 3. Results

### 3.1. HHcy Is Positively Linked to Cardiac Hypertrophy in Hypertensive Patients

To explore whether plasma Hcy level contributes to hypertensive cardiac hypertrophy, we first performed a retrospective analysis of 2101 inpatients with primary hypertension hospitalized in the First Affiliated Hospital of Dalian Medical University. These patients were divided into hypertrophy (783, 37.3%) and nonhypertrophy (1 318, 62.7%) cohorts, based on their echocardiographic evaluations. The demographic and clinical characteristics of these patients are presented in Table 1. Compared with patients in the nonhypertrophy group, those in the hypertrophy group were older and contained fewer males and smokers but had a higher prevalence of HHcy, type 2 diabetes mellitus (T_2_DM), coronary artery disease (CAD), chronic heart failure (CHF), dyslipidemia, and left atrial enlargement (LAE) (Table 1). Moreover, patients in the hypertrophy group had higher systolic blood pressure (SBP), diastolic blood pressure (DBP), and lower estimated glomerular filtration rate (eGFR) (Table 1). However, no significant difference in terms of drinking and the prevalence of atrial fibrillation (AF) as well as moderate and severe left ventricular systolic dysfunction was observed between these two cohorts (Table 1).  Table 2 shows the results of the univariate and multivariate logistic analysis of these two groups. HHcy (OR = 1.293; 95% CI: 1.049-1.594, *p* = 0.016) was found to be significantly associated with the presence of left ventricular hypertrophy (LVH) in these hypertensive patients, after adjusting for other conventional risk factors such as age, gender, smoke, CAD, CHF, T_2_DM, dyslipidemia, blood pressure, renal insufficiency, and LAE (Table 2). To further define the correlation of circulating Hcy levels with the risk of hypertensive LVH, we performed a quartile analysis based on serum Hcy levels. The respective cut-offs of serum Hcy levels for Q1, Q2, Q3, and Q4 were ≤11.67, 11.67-14.56, 14.56-18.61, and >18.61 *μ*mol/L, respectively. As shown in Table 3, the prevalence of LVH increased with quartiles of Hcy, and patients categorized into the Q4 quartile had the highest prevalence of LVH (Q1: 33.0%, Q2: 36.9%, Q3: 39.0%, and Q4: 40.2%) (Table 3). Compared with the risk of LVH in patients of the Q1 quartile, the ORs (95% CI) for LVH in the Q2, Q3, and Q4 quartiles were 1.289 (0.978-1.700, *p* = 0.072), 1.382 (1.034-1.848, *p* = 0.029), and 1.550 (1.141-2.106, *p* = 0.005), respectively (Table 3). Together, results from the retrospective analysis indicated that HHcy was positively linked with cardiac hypertrophy in hypertension.

### 3.2. HHcy Exacerbates AngII-Induced Cardiac Hypertrophy in Mice

**Figure 1:**
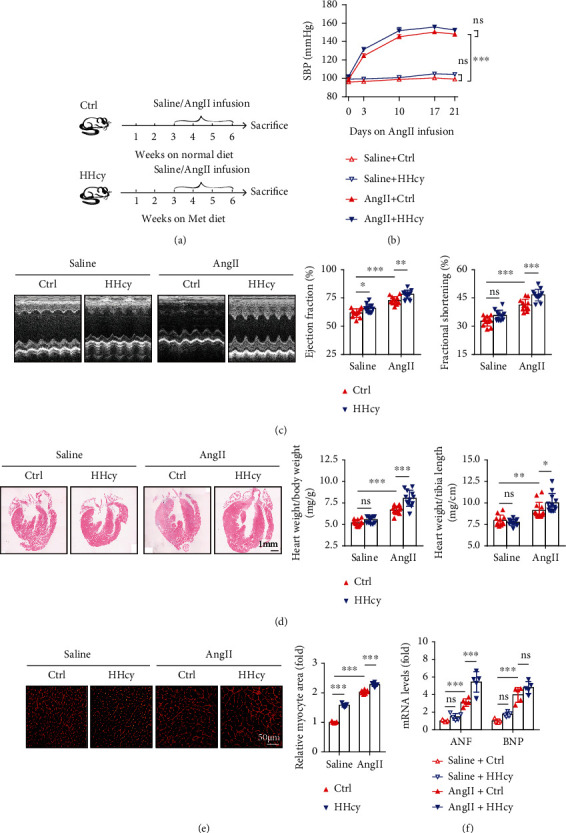
HHcy exacerbated AngII-induced cardiac hypertrophy in mice. (a) Schematic illustration of the experimental design. (b) Systolic blood pressure (SBP) collected before and at days 3, 10, 17, and 21 after AngII infusion, *n* = 12 − 13 per group. (c) Representative M-mode echocardiography at the end of the experiment and calculation of EF and FS, *n* = 12 − 13 per group. (d) Representative H&E staining of the heart sections and ratios of heart weight/body weight and heart weight/tibial length, *n* = 12 − 13 per group. (e) Representative WGA staining of the heart sections and quantitation of myocyte cross-sectional area, *n* = 5 − 6 per group. (f) Relative mRNA levels of ANF and BNP in the hearts, *n* = 5 − 6 per group. ^∗^*p* < 0.05, ^∗∗^*p* < 0.01, and ^∗∗∗^*p* < 0.001; ns: not significant.

We next explored the effect of HHcy on experimental cardiac hypertrophy in hypertensive mice. HHcy was induced by a Met (2% wt/wt) diet feeding as previously described [13, 14]. After being fed on this Met diet or control rodent chow diet for three weeks, mice were then subjected to AngII infusion at a dosage of 1000 ng/kg/min for another three weeks to induce hypertension and cardiac hypertrophy, and those receiving saline infusion were used as controls (Figure 1(a)). Our data showed that the Met diet feeding increased the plasma Hcy concentration to the level of mild HHcy (Figure S1). The presence of HHcy did not significantly exacerbate AngII-induced hypertension (Figure 1(b)) but strengthened cardiac contractility, as reflected by a prominent increase of left ventricular (LV) ejection fraction (EF) and fractional shortening (FS) in echocardiography (Figure 1(c)). HHcy also exacerbated AngII-induced heart enlargement, as indicated by gross morphological H&E staining, increased ratio of heart weight/body weight and heart weight/tibial length (Figure 1(d)) as well as increased cross-sectional area of myocytes shown by WGA staining (Figure 1(e)). Moreover, HHcy increased AngII-stimulated cardiac expression of atrial natriuretic factor (ANF) and brain natriuretic peptide (BNP), two markers widely used to evaluate cardiac injury and dysfunction, although the increase of BNP did not reach statistical significance due to the relatively small sample size and large within-group variation (Figure 1(f)). Notably, it seemed that HHcy exerted a mild pro-hypertrophic response on cardiomyocytes even without AngII stimulation, as shown by an increase of myocyte cross-sectional area (Figure 1(e)) and a nonsignificant increase of cardiac ANF and BNP expression (Figure 1(f)), which might be too weak to reach functional or gross-morphological significance (Figures 1(c) and 1(d)).

### 3.3. HHcy Exacerbates AngII-Induced Cardiac Inflammation and Fibrosis in Mice

**Figure 2:**
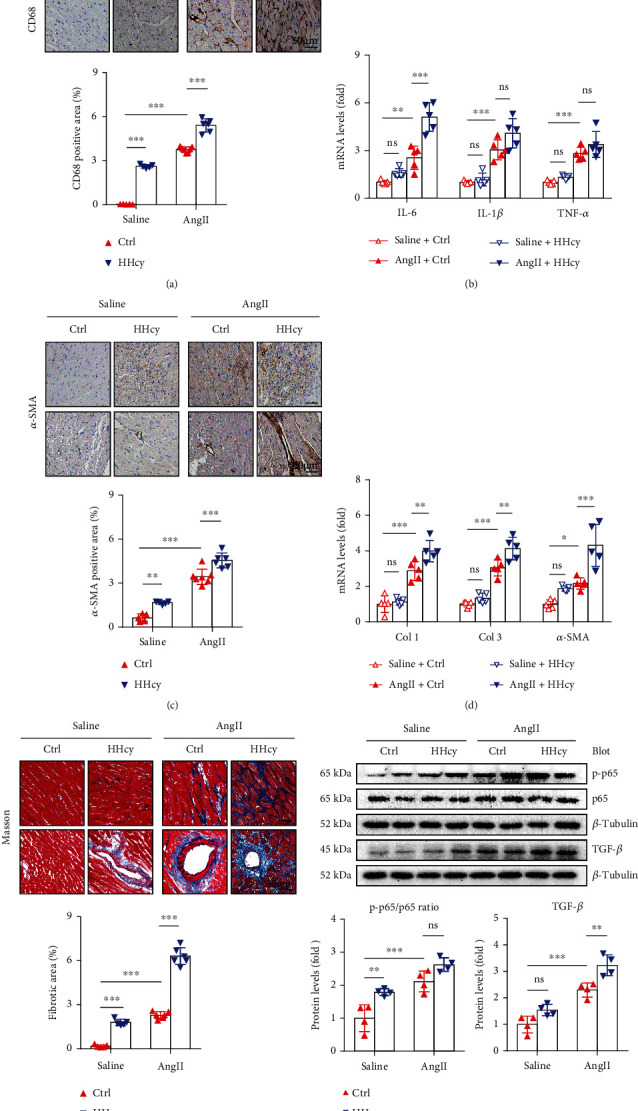
HHcy exacerbated AngII-induced cardiac inflammation and fibrosis in mice. (a) Representative H&E and CD68 immunohistochemical staining of the heart sections and quantitation of CD68-positive fraction, *n* = 5 per group. (b) Relative mRNA levels of IL-6, IL-1*β*, and TNF-*α*, *n* = 5 per group. (c) Representative *α*-SMA immunohistochemical staining of the heart sections and quantitation of *α*-SMA-positive fraction, *n* = 5 − 7 per group. (d) Relative mRNA levels of Col 1, Col 3, and *α*-SMA, *n* = 5 per group. (e) Representative Masson staining of the heart sections and quantitation of fibrotic fraction, *n* = 5 − 6 per group. (f) Representative Western blots and quantitation of NF-*κ*B p-p65/p65 and TGF-*β*, *n* = 4 per group. ^∗^*p* < 0.05, ^∗∗^*p* < 0.01, and ^∗∗∗^*p* < 0.001; ns: not significant.

Pathological cardiac hypertrophy is often in companion with cardiac inflammation and fibrosis [1]. Using H&E and CD68 immunohistochemical staining, we showed that HHcy significantly exacerbated AngII-induced inflammatory cell (mainly macrophage) infiltration into the myocardium (Figure 2(a)). The proinflammatory effect of HHcy was further conformed by detecting the cardiac gene expression level of proinflammatory cytokines, such as IL-6, IL-1*β*, and TNF-*α*, using quantitative real-time PCR. As shown in Figure 2(b), HHcy exacerbated AngII-induced increase of inflammatory cytokine expression, although the increase of IL-1*β* and TNF-*α* expression did not reach statistical significance based on the limited sample size (Figure 2(b)). In addition to cardiac inflammation, AngII infusion also stimulated fibroblast cell proliferation and expression of collagen I (Col1) and collagen III (Col3), finally contributing to cardiac fibrosis. Using *α*-SMA immunochemical staining and quantitative real-time PCR, we showed that HHcy exacerbated AngII-stimulated proliferation of *α*-SMA-positive fibroblasts (Figure 2(c)) as well as gene expression of *α*-SMA, Col1 and Col3 (Figure 2(d)), finally leading to increased interstitial and perivascular fibrosis shown by Masson staining (Figure 2(e)). As NF-*κ*B and TGF-*β* signaling are key pathways regulating AngII-induced cardiac inflammation and fibrosis, we detected these pathways by Western blot. Our data showed that HHcy further enhanced AngII-induced NF-*κ*B p65 and TGF-*β* activation (Figure 2(f)), suggesting that HHcy exacerbated AngII-induced cardiac inflammation and fibrosis at least partially through these two pathways. Interestingly, HHcy itself was capable to induce mild cardiac inflammation and fibrosis, as shown by histological staining (Figures 2(a), 2(c), and 2(e)), and these two signaling pathways might also be involved (Figure 2(f)).

### 3.4. FA Supplement Attenuates AngII-Induced Cardiac Hypertrophy in Hyperhomocysteinemic Mice

**Figure 3:**
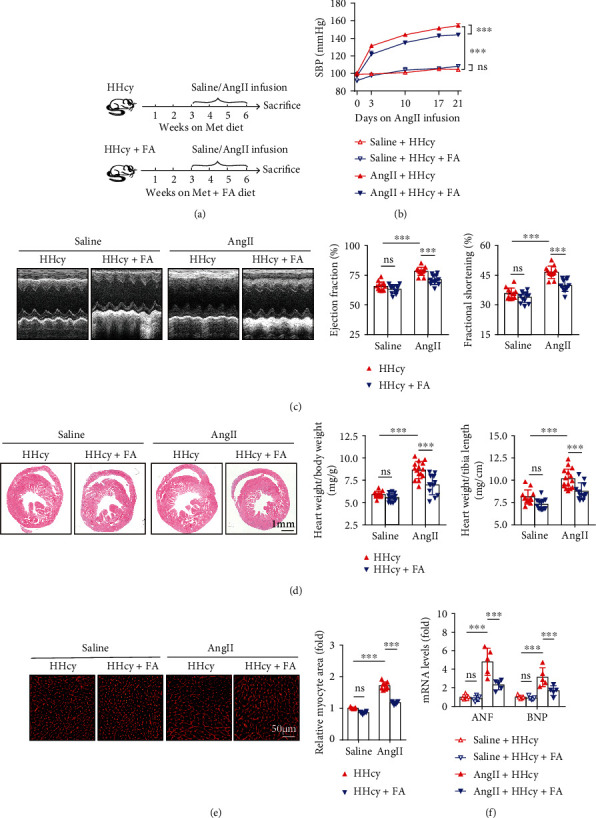
FA supplement attenuated HHcy-aggravated cardiac hypertrophy in mice. (a) Schematic illustration of the experimental design. (b) Systolic blood pressure (SBP) collected before and at days 3, 10, 17, and 21 after AngII infusion, *n* = 13 per group. (c) Representative M-mode echocardiography at the end of the experiment and calculation of EF and FS, *n* = 13 per group. (d) Representative H&E staining of the heart sections and ratios of heart weight/body weight and heart weight/tibial length, *n* = 13 per group. (e) Representative WGA staining of the heart sections and quantitation of myocyte cross-sectional area, *n* = 5 − 7 per group. (f) Relative mRNA levels of ANF and BNP in the hearts, *n* = 5 per group. ^∗∗∗^*p* < 0.001; ns: not significant.

FA is demonstrated to be effective to reverse HHcy [22]. To explore the therapeutic potent of FA supplement in HHcy-stimulated cardiac hypertrophy, we fed mice with FA-supplemented Met diet during the experimental process (Figure 3(a)). We found that FA (0.006% wt/wt) supplement corrected diet-induced HHcy (Figure S2) and significantly inhibited AngII-induced increase of blood pressure (Figure 3(b)) and cardiac contractility (Figure 3(c)) in hyperhomocysteinemic mice. FA supplement also attenuated AngII-induced heart enlargement, as reflected by gross morphological staining as well as a decreased ratio of heart weight/body weight and heart weight/tibia length (Figure 3(d)). Moreover, FA supplement attenuated AngII-induced increase of myocyte cross-sectional area (Figure 3(e)) and cardiac ANF and BNP gene expression (Figure 3(f)) in hyperhomocysteinemic mice.

### 3.5. FA Supplement Attenuates AngII-Induced Cardiac Inflammation and Fibrosis in Hyperhomocysteinemic Mice

**Figure 4:**
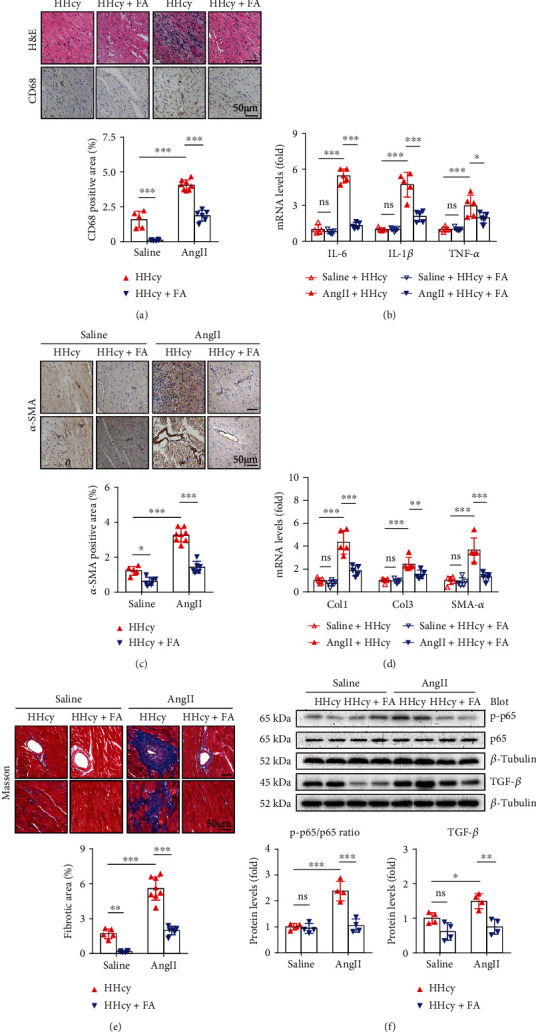
FA supplement attenuated HHcy-aggravated cardiac inflammation and fibrosis in mice. (a) Representative H&E and CD68 immunohistochemical staining of the heart sections and quantitation of CD68-positive fraction, *n* = 5 − 7 per group. (b) Relative mRNA levels of IL-6, IL-1*β*, and TNF-*α*, *n* = 5 per group. (c) Representative *α*-SMA immunohistochemical staining of the heart sections and quantitation of *α*-SMA-positive fraction, *n* = 5 − 8 per group. (d) Relative mRNA levels of Col 1, Col 3, and *α*-SMA, *n* = 5 per group. (e) Representative Masson staining of the heart sections and quantitation of fibrotic fraction, *n* = 5 − 8 per group. (f) Representative Western blots and quantitation of NF-*κ*B p-p65/p65 and TGF-*β*, *n* = 4 per group. ^∗^*p* < 0.05, ^∗∗^*p* < 0.01, and ^∗∗∗^*p* < 0.001; ns: not significant.

We then explored the effects of FA supplement on cardiac inflammation and fibrosis. Our data suggested that FA supplement significantly inhibited AngII-induced inflammatory cell infiltration (Figure 4(a)) as well as the expression of proinflammatory cytokines (Figure 4(b)). Likewise, FA supplement significantly inhibited AngII-stimulated fibroblast activation, demonstrated by reduced *α*-SMA-positive fibroblast proliferation (Figure 4(c)) and decreased expression of *α*-SMA, Col1 and Col3 (Figure 4(d)), as well as reduced interstitial and perivascular fibrosis (Figure 4(e)). Moreover, FA also reduced cardiac inflammation and fibrosis in hyperhomocysteinemic mice without AngII stimulation (Figures 4(a), 4(c), and 4(e)). These protective effects of FA supplement on cardiac inflammation and fibrosis were accompanied by reduced NF-*κ*B and TGF-*β* signaling (Figure 4(f)).

### 3.6. Calcineurin-NFAT Signaling Might Be Involved in the Pro-hypertrophic Effect of Hcy

**Figure 5:**
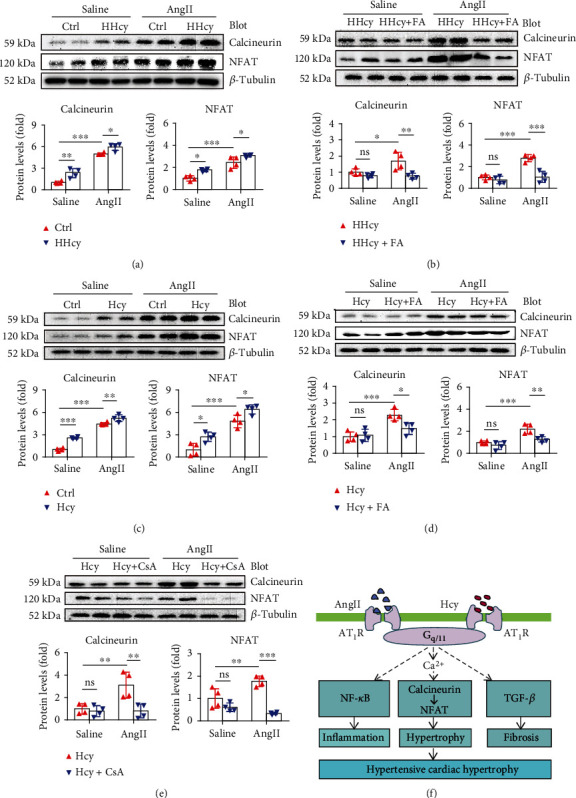
Calcineurin-NFAT signaling might be involved in the pro-hypertrophic effect of Hcy. (a) Representative Western blot and quantitation of cardiac Calcineurin and NFAT in hyperhomocysteinemic mice. *n* = 4 per group. (b) Representative Western blot and quantitation of cardiac Calcineurin and NFAT in hyperhomocysteinemic mice treated with FA. *n* = 4 per group. (c) Representative Western blots and quantitation of Calcineurin and NFAT in Hcy-treated NRCMs. *n* = 4 per group. (d) Representative Western blots and quantitation of Calcineurin and NFAT in FA-treated NRCMs. *n* = 4 per group. (e) Representative Western blot and quantitation of Calcineurin and NFAT in CsA-treated NRCMs. *n* = 4 per group. (f) A working model of Hcy in AngII-induced hypertensive cardiac hypertrophy. ^∗^*p* < 0.05, ^∗∗^*p* < 0.01, and ^∗∗∗^*p* < 0.001; ns: not significant.

Calcineurin-NFAT signaling is a classic pathway regulating cardiac hypertrophy [23, 24]. To explore whether this signaling pathway is involved in the pathogenesis of HHcy in cardiac hypertrophy, we first detected the expression of Calcineurin and NFAT by Western blot in AngII-stimulated mice. Our data showed that both Calcineurin and NFAT expressions were increased by Met-induced HHcy (Figure 5(a)) and could be eased by FA supplement (Figure 5(b)). In cultured NRCMs, Hcy treatment significantly exacerbated AngII-induced cardiomyocyte hypertrophy, as visualized by immunofluorescence staining using myocardial-specific a-actinin (Figure S3), which was associated with enhanced Calcineurin and NFAT expression (Figure 5(c)) and could be significantly attenuated by FA supplement (Figure S4 and 5D). Interestingly, inhibition of Calcineurin-NFAT signaling with Calcineurin inhibitor CsA attenuated Hcy-exacerbated cardiomyocyte hypertrophy (Figure 5(e) and S5).

## 4. Discussion

In this study, by retrospectively analyzing a cohort of hypertensive patients and exploring AngII-induced cardiac hypertrophy models, we show that (1) the prevalence of HHcy is higher in patients with hypertrophy and significantly associated with the presence of cardiac hypertrophy after adjusting for other conventional risk factors in hypertensive patients; (2) Met-induced HHcy increases AngII-stimulated cardiac hypertrophy as well as inflammation and fibrosis; (3) the pathogenic effects of HHcy on AngII-induced cardiac phenotypes could be partially reversed by FA supplement; and (4) Calcineurin-NFAT signaling might be involved in the pathogenesis of Hcy. The working model of Hcy in AngII-induced hypertensive cardiac hypertrophy is illustrated in Figure 5(f).

Hypertension attacks 1.39 billion adults, that is, more than 30% of the world's total adults in 2010 [25]. Unfortunately, about half (53.5%) of them are unaware of their condition, and less than 14% of them effectively control their blood pressure after antihypertension medication [25]. Recently, a cross-sectional study involving 11,007 participants showed that more than one-third (36.1%) of hypertensive patients comorbid with HHcy [26]; therefore, elucidation of the pathogenesis of HHcy in hypertension and hypertension-associated diseases, such as hypertensive cardiac hypertrophy and atrial fibrillation, is of pivotal significance for the clinical prevention and treatment of the diseases. As an established risk factor for hypertension, HHcy is positively correlated with blood pressure. An increase of 5 *μ*mol/L of circulating HHcy concentration is estimated to increase systolic and diastolic blood pressure by 0.5-0.7 mmHg and 0.7-1.2 mmHg, respectively [27]. Hcy promotes hypertension through multiply mechanisms, involving endothelial dysfunction, intima-media thickening, and adventitial inflammation, caused by oxidative stress, protein modification, inflammation activation, proliferation of vascular smooth muscle cells, and inhibition of fibrinolysis [28]. In the current study, we observed a mild increase of blood pressure after AngII stimulation in the Met-fed hyperhomocysteinemic mice, compared with the chow-fed control mice. Although such increases of blood pressure did not reach statistical significance, it might partially mediate HHcy's pro-hypertrophic effect in AngII-induced hypertensive cardiac hypertrophy, due to a direct increase of cardiac workload.

The correlation between HHcy and the prevalence of pathological cardiac hypertrophy has been explored as early as 20 years ago, when a cross-sectional study for the first time showed a positive association between plasma Hcy concentration and the risk of developing cardiac hypertrophy in a limited 75 end-stage renal disease patients undergoing chronic hemodialysis [29]. Later, in a large community-based Framingham Heart Study involving 2 697 Caucasian-dominant participants, circulating Hcy concentrations were found significantly related to pathological cardiac hypertrophy in females but not in males [30]. Recently, a Chinese cohort study also indicated that HHcy independently promotes cardiac hypertrophy and the prohypertrophic effect of HHcy is prominent in both genders and could be enhanced in combination with metabolic syndrome [31]. Here, we show that among 2101 Chinese hypertensive patients, those who develop hypertensive cardiac hypertrophy have a higher prevalence of HHcy, and a statistical link between HHcy and the presence of cardiac hypertrophy is identified, after adjusting for other conventional risk factors such as age, gender, smoke, and CAD. Therefore, data from different ethnic groups and different disease conditions all suggest that HHcy might be a potential risk factor for human pathological cardiac hypertrophy.

In addition to clinical observational studies, experimental evidence also indicates a potential causative role of HHcy in pathological cardiac hypertrophy. In a normotensive rat model, cardiac hypertrophy can be observed after 8-week Met diet feeding, although the underlying mechanisms are still unclear [32, 33]. Interestingly, valsartan, an antagonist of angiotensin type 1 receptor (AT_1_R), significantly attenuates HHcy-induced cardiac hypertrophy in normotension, indicating an involvement of AT_1_R activation in HHcy's pathogenic effects [34]. Unlike normotension, the renin-angiotensin-aldosterone system is overactivated in hypertension, which leads to an increase of systemic production of AngII [35]. The binding of AngII to AT_1_R then initiates several key intracellular signaling pathways, finally leading to cardiovascular consequences such as cardiac hypertrophy and aortic aneurysm [36]. In addition to AngII, AT_1_R can be also activated by other stimuli, including mechanical stretch and AT_1_R autoantibodies [36]. Recently, Hcy is demonstrated to be another agonist of AT_1_R and capable of directly binding to AT_1_R by forming a salt bridge and a disulfide bond with the Arg167 and Cys289 residues of AT_1_R, indicating that Hcy and AngII could synergistically activate the AT_1_R [36]. Although the binding affinity of Hcy to AT_1_R is significantly lower (10^5^ times) than that of AngII, the circulating Hcy concentration, especially in HHcy condition, is however much higher (~5 log magnitude) than that of AngII [36]; therefore, Hcy-induced AT_1_R activation might add up the effects of AngII-AT_1_R activation in HHcy, finally promoting AngII-induced cardiac hypertrophy.

As a G-protein-coupled receptor, AT_1_R activation by binding with ligands leads to subsequent activation of G proteins such as G_q/11_ [1]. Activation of G_q/11_ signaling then activates phospholipase C and inositol 1,4,5-triphosphate (IP_3_) synthesis, inducing intracellular Ca^2+^ release from endoplasmic and sarcoplasmic reticulum [1]. Increase of intracellular Ca^2+^ further activates Calcineurin, a Ca^2+^-activated serine/threonine protein phosphatase, which dephosphorylates and promotes nuclear localization of NFAT, finally activating downstreamed transcription factors such as GATA binding protein 4 and myocyte enhancer factor 2 to regulate hypertrophic response [23, 24]. In the current study, we observed an additional increase of AngII-stimulated Calcineurin and NFAT expression in hyperhomocysteinemic mice and Hcy-treated NRCMs, which could be attenuated by FA both *in vivo* and *in vitro*. Interestingly, inhibition of Calcineurin-NFAT pathway by CsA, an inhibitor of Calcineurin, abolishes Hcy-aggravated NRCM hypertrophy *in vitro*. These data together suggest that Calcineurin-NFAT signaling might be involved in the pathogenesis of Hcy in hypertensive cardiac hypertrophy.

FA, also known as vitamin B9, mediates the remethylation of Hcy into Met by acting as a cofactor of methylenetetrahydrofolate reductase, therefore playing an important role in maintaining Hcy homeostasis [5]. FA has been demonstrated to be effective in reducing circulating Hcy levels in both clinical and experimental studies. In addition to its Hcy-lowering effect, FA, as a member of the vitamin B family, has been suggested to have other cardiovascular benefits. For example, FA improves cardiac remodeling and function in aging [37] and doxorubicin-induced cardiotoxicity [38, 39], by reducing cardiac senescence, apoptosis, oxidative stress, and fibrosis. A combined use of cobalamin (vitamin B12) and FA preserves cardiac mitochondrial function and biogenesis in pressure-overloaded mice [40]; moreover, FA supplement is capable to reduce endocardial endothelial dysfunction in homocysteinemic hypertensive rats [41]. Here, we show that FA supplement corrects HHcy and remarkably attenuates AngII-induced cardiac hypertrophy as well as cardiac inflammation and fibrosis in hyperhomocysteinemic mice, alongside with a significant inhibition of AngII-induced increase of blood pressure. Interestingly, the antihypertrophic effect of FA is also observed in NRCMs subjected to AngII and Hcy stimulation, indicating a direct and pressure-independent protection against hypertensive cardiac hypertrophy, which might be closely associated with Calcineurin-NFAT signaling as demonstrated in the current study. Unfortunately, participants enrolled in the retrospective study did not take FA as a routine medication against HHcy; we herein are unable to provide further clinical data to support the therapeutic potent of FA in HHcy-stimulated hypertensive cardiac hypertrophy.

In conclusion, our study indicates that HHcy might be a potential risk factor for cardiac hypertrophy in hypertension, and Calcineurin-NFAT pathway might be involved in the pro-hypertrophic effect of Hcy.

## Figures and Tables

**Table 1:** Baseline characteristics of the participants in the cohort study.

Variables	Cohort		*p* value
	Nonhypertrophy (*n* = 1318)	Hypertrophy (*n* = 783)	
Age (years)	67 (61-74)	68 (62-77)	0.001
Male sex, *n* (%)	874 (66.3)	444 (56.7)	<0.001
Smoke, *n* (%)	437 (33.2)	213 (27.2)	0.004
Drink, *n* (%)	275 (20.9)	144 (18.4)	0.170
SBP (mmHg)	140 (130-155)	146 (135-160)	<0.001
DBP (mmHg)	80 (75-90)	82 (78-91)	0.010
Pulse (beats/min)	72 (68-80)	72 (68-80)	0.481
*Medical history*			
T_2_DM, *n* (%)	408 (31.0)	324 (41.4)	<0.001
AF, *n* (%)	243 (18.4)	161 (20.6)	0.232
CAD, *n* (%)	376 (28.5)	260 (33.2)	0.024
CHF, *n* (%)	103 (7.8)	85 (10.9)	0.018
*Laboratory data*			
eGFR (mL/(min•1.73 m^2^))	90.0 (74.0-105.0)	86.0 (67.0-102.0)	<0.001
eGFR < 90 (mL/(min•1.73 m^2^))	637 (48.3)	426 (54.4)	0.007
TC (mmol/L)	4.6 (3.8-5.4)	4.7 (3.9-5.5)	0.017
TG (mmol/L)	1.3 (1.0-1.7)	1.3 (1.0-1.8)	0.002
HDL-C (mmol/L)	1.1 (1.0-1.4)	1.1 (0.9-1.3)	0.199
LDL-C (mmol/L)	2.5 (2.0-3.1)	2.6 (2.1-3.1)	0.003
Dyslipidemia, *n* (%)	633 (48.0)	419 (53.5)	0.015
Hcy (*μ*mol/L)	14.4 (11.5-18.3)	15.0 (12.2-18.9)	0.010
HHcy, *n* (%)	582 (44.2)	392 (50.1)	0.009
*Echocardiographic parameters*			
LAD (mm)	36.0 (33.0-39.0)	39.0 (36.0-43.0)	<0.001
LAE, *n* (%)	271 (20.6)	328 (41.9)	<0.001
LVEDD (mm)	46.0 (42.0-49.0)	46.0 (43.0-49.0)	0.287
LVEF (%)	57.0 (55.0-59.0)	57.0 (55.0-58.0)	0.002
LVEF < 40%, *n* (%)	40 (3.0)	25 (3.2)	0.840
IVST (mm)	10.0 (9.0-10.5)	12.0 (12.0-13.0)	<0.001
LVPWT (mm)	10.0 (9.0-10.0)	11.0 (11.0-12.0)	<0.001
LVM (g)	153.3 (132.8-180.4)	200.5 (175.0-232.2)	<0.001
*Medication*			
Antihypertensive use, *n* (%)	1179 (89.5)	728 (93.0)	0.007
ACEI/ARB, *n* (%)	588 (44.6)	421 (53.8)	<0.001
*β*-Blocker, *n* (%)	471 (35.7)	335 (42.8)	0.001
CCB, *n* (%)	854 (64.8)	571 (72.9)	<0.001
Diuretic, *n* (%)	485 (36.8)	353 (45.1)	<0.001

Abbreviation: ACEI: angiotensin-converting enzyme inhibitors; AF: atrial fibrillation; ARB: angiotensin-converting enzyme receptor blockers; CAD: coronary artery disease; CCB: calcium channel blockers; CHF: chronic heart failure; DBP: diastolic blood pressure; eGFR: estimated glomerular filtration rate; SBP: systolic blood pressure; Hcy: homocysteine; HDL-C: high-density lipoprotein cholesterol; HHcy: hyperhomocysteinemia; IVST: interventricular septal thickness; LAD: left atrium diameter; LAE: left atrial enlargement; LDL-C: low-density lipoprotein cholesterol; LVEDD: left ventricular end diastolic diameter; LVEF: left ventricular ejection fraction; LVM: left ventricular mass; LVPWT: left ventricular posterior wall thickness; T_2_DM: type 2 diabetes mellitus; TC: total cholesterol; TG: triglycerides.

**Table 2:** Risk factors associated with cardiac hypertrophy in the cohort study.

Variable	Univariate analysis		Multivariate analysis	
	OR (95% CI)	*p* value	OR (95% CI)	*p* value
Age (years)	1.013 (1.005-1.022)	0.002	1.002 (0.992-1.012)	0.678
Male sex	0.665 (0.555-0.798)	<0.001	0.649 (0.518-0.813)	<0.001
Smoke	0.753 (0.620-0.915)	0.004	0.944 (0.746-1.195)	0.634
Drink	0.855 (0.683-1.070)	0.170		
SBP (mmHg)	1.013 (1.009-1.018)	<0.001	1.011 (1.006-1.016)	<0.001
DBP (mmHg)	1.011 (1.004-1.018)	0.001	1.004 (0.996-1.012)	0.294
Pulse (beats/min)	0.999 (0.993-1.005)	0.757		
*Medical history*				
T_2_DM	1.574 (1.310-1.892)	<0.001	1.458 (1.194-1.780)	<0.001
AF	1.145 (0.917-1.430)	0.232		
CAD	1.245 (1.029-1.507)	0.024	1.128 (0.918-1.387)	0.251
CHF	1.436 (1.062-1.943)	0.019	0.837 (0.600-1.169)	0.297
*Laboratory data*				
eGFR < 90 (mL/(min•1.73 m^2^))	1.276 (1.068-1.523)	0.007	1.049 (0.855-1.286)	0.649
Dyslipidemia	1.246 (1.043-1.487)	0.015	1.250 (1.032-1.514)	0.022
HHcy	1.268 (1.062-1.514)	0.009	1.293 (1.049-1.594)	0.016
*Echocardiographic parameters*				
LAE	2.785 (2.292-3.385)	<0.001	2.663 (2.158-3.286)	<0.001
LVEDD, mm	1.010 (0.993-1.026)	0.251		
LVEF < 40%	1.054 (0.634-1.751)	0.840		
*Medication*				
Antihypertensive use	1.561 (1.127-2.161)	0.007	1.197 (0.851-1.685)	0.302

Abbreviations as in Table 1.

**Table 3:** Risk of cardiac hypertrophy according to serum Hcy quartiles.

	Patients	Patients with hypertrophy (%)	OR (95% CI)
Q1	525	173 (33.0%)	1 (ref.)
Q2	526	194 (36.9%)	1.289 (0.978-1.700)
Q3	528	206 (39.0%)	1.382 (1.034-1.848)^∗^
Q4	522	210 (40.2%)	1.55 (1.141-2.106)^∗∗^

^∗^ *p* < 0.05 and ^∗∗^*p* < 0.01.

## Data Availability

The data used to support the findings of this study are included within the article.

## Abbreviations

ANF: Atrial natriuretic factor
AngІІ: Angiotensin ІІ
AT_1_R: Angiotensin type 1 receptor
BNP: Brain natriuretic peptide
CsA: Cyclosporin A
EF: Ejection fraction
FA: Folic acid
FS: Fractional shortening
Hcy: Homocysteine
HHcy: Hyperhomocysteinemia
Met: Methionine
NFAT: Nuclear factor of activated T cells
NF-*κ*B: Nuclear transcription factor-*κ*B
NRCMs: Neonatal rat cardiomyocytes
TGF-*β*: Transforming growth factor-*β*
WGA: Wheat germ agglutinin.

## Supplementary Materials

Supplementary Materials Table S1: List of primers used in the study. Figure S1: Methionine (Met) diet induced a mild HHcy in mice. Figure S2: FA supplement corrected Met diet-induced HHcy in mice. Figure S3: Hcy exacerbated AngII-induced pro-hypertrophic response in NRCMs. Figure S4: FA supplement attenuated HHcy-stimulated pro-hypertrophic response in NRCMs. Figure S5: CsA attenuated HHcy-stimulated pro-hypertrophic response in NRCMs.

## References

[1] Nakamura M., Sadoshima J. (2018). Mechanisms of physiological and pathological cardiac hypertrophy. *Nature Reviews. Cardiology*.

[2] Kumar A., Palfrey H. A., Pathak R., Kadowitz P. J., Gettys T. W., Murthy S. N. (2017). The metabolism and significance of homocysteine in nutrition and health. *Nutrition and Metabolism*.

[3] Zaric B. L., Obradovic M., Bajic V., Haidar M. A., Jovanovic M., Isenovic E. R. (2019). Homocysteine and hyperhomocysteinaemia. *Current Medicinal Chemistry*.

[4] Clarke R., Daly L., Robinson K. (1991). Hyperhomocysteinemia: an independent risk factor for vascular disease. *The New England Journal of Medicine*.

[5] Fu Y., Wang X., Kong W. (2018). Hyperhomocysteinaemia and vascular injury: advances in mechanisms and drug targets. *British Journal of Pharmacology*.

[6] Yao G. H., Deng Y., Liu Y. (2015). Echocardiographic measurements in normal Chinese adults focusing on cardiac chambers and great arteries: a prospective, nationwide, and multicenter study. *Journal of the American Society of Echocardiography*.

[7] American Diabetes Association (2018). 2. Classification and diagnosis of diabetes: standards of medical care in diabetes—2018. *Diabetes care*.

[8] Lassen M. C. H., Skaarup K. G., Lind J. N., Alhakak A. S., Sengelov M., Nielsen A. B. (2020). Echocardiographic abnormalities and predictors of mortality in hospitalized COVID-19 patients: the ECHOVID-19 study. *ESC heart failure*.

[9] Ji C., Kaplowitz N. (2004). Hyperhomocysteinemia, endoplasmic reticulum stress, and alcoholic liver injury. *World journal of gastroenterology: WJG*.

[10] Developed with the Special Contribution of the European Heart Rhythm Association (EHRA), Endorsed by the European Association for Cardio-Thoracic Surgery (EACTS), Authors/Task Force Members, Camm A. J., Kirchhof P. (2010). Guidelines for the management of atrial fibrillation: the task force for the management of atrial fibrillation of the European Society of Cardiology (ESC). *European Heart Journal*.

[11] Lang R. M., Badano L. P., Mor-Avi V. (2015). Recommendations for cardiac chamber quantification by echocardiography in adults: an update from the American Society of Echocardiography and the European Association of Cardiovascular Imaging. *European Heart Journal-Cardiovascular Imaging*.

[12] National K. F. (2002). K/DOQI clinical practice guidelines for chronic kidney disease: evaluation, classification, and stratification. *American Journal of Kidney Diseases*.

[13] Zhang D. H., Chen Y. Q., Xie X. N. (2012). Homocysteine activates vascular smooth muscle cells by DNA demethylation of platelet-derived growth factor in endothelial cells. *Journal of Molecular and Cellular Cardiology*.

[14] Ai Y. B., Sun Z. Z., Peng C., Liu L. L., Xiao X. Q., Li J. B. (2017). Homocysteine induces hepatic steatosis involving ER stress response in high methionine diet-fed mice. *Nutrients*.

[15] Li M., Chen J., Li Y. S., Feng Y. B., Gu X., Shi C. Z. (2006). Folic acid reduces adhesion molecules VCAM-1 expression in aortic of rats with hyperhomocysteinemia. *International Journal of Cardiology*.

[16] Li M., Chen J., Li Y. S., Feng Y. B., Zeng Q. T. (2007). Folic acid reduces chemokine MCP-1 release and expression in rats with hyperhomocystinemia. *Cardiovascular Pathology*.

[17] Wang L., Zhang Y.-L., Lin Q.-Y. (2018). CXCL1–CXCR2 axis mediates angiotensin II-induced cardiac hypertrophy and remodelling through regulation of monocyte infiltration. *European Heart Journal*.

[18] Wang L., Zhao X. C., Cui W. (2016). Genetic and pharmacologic inhibition of the chemokine receptor CXCR2 prevents experimental hypertension and vascular dysfunction. *Circulation*.

[19] Liao J., Guo X., Wang M. (2017). Scavenger receptor class B type 1 deletion led to coronary atherosclerosis and ischemic heart disease in low-density lipoprotein receptor knockout mice on modified Western-type diet. *Journal of Atherosclerosis and Thrombosis*.

[20] Li H.-H., Kedar V., Zhang C. (2004). Atrogin-1/muscle atrophy F-box inhibits calcineurin-dependent cardiac hypertrophy by participating in an SCF ubiquitin ligase complex. *The Journal of Clinical Investigation*.

[21] Liao J. W., An X. B., Yang X. L. (2020). Deficiency of LMP10 attenuates diet-induced atherosclerosis by inhibiting macrophage polarization and inflammation in apolipoprotein E deficient mice. *Frontiers in Cell and Development Biology*.

[22] Jacques P. F., Selhub J., Bostom A. G., Wilson P. W., Rosenberg I. H. (1999). The effect of folic acid fortification on plasma folate and total homocysteine concentrations. *The New England Journal of Medicine*.

[23] Parra V., Rothermel B. A. (2017). Calcineurin signaling in the heart: the importance of time and place. *Journal of Molecular and Cellular Cardiology*.

[24] Creamer T. P. (2020). Calcineurin. *Cell Communication and Signaling: CCS*.

[25] Mills K. T., Stefanescu A., He J. (2020). The global epidemiology of hypertension. *Nature Reviews. Nephrology*.

[26] Cheng M. N., Xue H., Li X. J. (2022). Prevalence of hyperhomocysteinemia (HHcy) and its major determinants among hypertensive patients over 35 years of age. *European Journal of Clinical Nutrition*.

[27] Lim U., Cassano P. A. (2002). Homocysteine and blood pressure in the Third National Health and Nutrition Examination Survey, 1988-1994. *American Journal of Epidemiology*.

[28] Ganguly P., Alam S. F. (2015). Role of homocysteine in the development of cardiovascular disease. *Nutrition Journal*.

[29] Blacher J., Demuth K., Guerin A. P. (1999). Association between plasma homocysteine concentrations and cardiac hypertrophy in end-stage renal disease. *Journal of Nephrology*.

[30] Sundström J., Sullivan L., Selhub J. (2004). Relations of plasma homocysteine to left ventricular structure and function: the Framingham Heart Study. *European Heart Journal*.

[31] Yu S. S., Chen Y. T., Yang H. M., Guo X. F., Zheng L. Q., Sun Y. X. (2020). Hyperhomocysteinemia accompany with metabolic syndrome increase the risk of left ventricular hypertrophy in rural Chinese. *BMC Cardiovascular Disorders*.

[32] Joseph J., Joseph L., Shekhawat N. S. (2003). Hyperhomocysteinemia leads to pathological ventricular hypertrophy in normotensive rats. *American Journal of Physiology. Heart and Circulatory Physiology*.

[33] Zhao Q., Song W., Huang J., Wang D., Xu C. (2021). Metformin decreased myocardial fibrosis and apoptosis in hyperhomocysteinemia -induced cardiac hypertrophy. *Current Research in Translational Medicine*.

[34] Kassab S., Garadah T., Abu-Hijleh M. (2006). The angiotensin type 1 receptor antagonist valsartan attenuates pathological ventricular hypertrophy induced by hyperhomocysteinemia in rats. *Journal of the Renin-Angiotensin-Aldosterone System*.

[35] Paz Ocaranza M., Riquelme J. A., García L. (2020). Counter-regulatory renin-angiotensin system in cardiovascular disease. *Nature Reviews. Cardiology*.

[36] Li T. Y., Yu B., Liu Z. X. (2018). Homocysteine directly interacts and activates the angiotensin II type I receptor to aggravate vascular injury. *Nature Communications*.

[37] Ye S., Zhou X., Chen P., Lin J. F. (2021). Folic acid attenuates remodeling and dysfunction in the aging heart through the ER stress pathway. *Life Sciences*.

[38] Ahmad S., Panda B. P., Kohli K., Fahim M., Dubey K. (2017). Folic acid ameliorates celecoxib cardiotoxicity in a doxorubicin heart failure rat model. *Pharmaceutical Biology*.

[39] Octavia Y., Kararigas G., de Boer M. (2017). Folic acid reduces doxorubicin-induced cardiomyopathy by modulating endothelial nitric oxide synthase. *Journal of Cellular and Molecular Medicine*.

[40] Piquereau J., Moulin M., Zurlo G. (2017). Cobalamin and folate protect mitochondrial and contractile functions in a murine model of cardiac pressure overload. *Journal of Molecular and Cellular Cardiology*.

[41] Miller A., Mujumdar V., Palmer L., Bower J. D., Tyagi S. C. (2002). Reversal of endocardial endothelial dysfunction by folic acid in homocysteinemic hypertensive rats. *American Journal of Hypertension*.

