# Supplementary material for: Hyperhomocysteinemia Promotes Cardiac Hypertrophy in Hypertension
Source: Oxid Med Cell Longev. 2022 Aug 22;2022:1486157. doi: 10.1155/2022/1486157 (PMC9423973; doi:10.1155/2022/1486157)
Supplement: Supplementary Materials — Table S1: List of primers used in the study. Figure S1: Methionine (Met) diet induced a mild HHcy in mice. Figure S2: FA supplement corrected Met diet-induced HHcy in mice. Figure S3: Hcy exacerbated AngII-induced pro-hypertrophic response in NRCMs. Figure S4: FA supplement attenuated HHcy-stimulated pro-hypertrophic response in NRCMs. Figure S5: CsA attenuated HHcy-stimulated pro-hypertrophic response in NRCMs. [file 1486157.f1.docx]

**Supplemental Materials and Methods**

**Serum Hcy measurement**

Blood was collected by retro-orbital bleeding. Serum Hcy was measured with a commercial Elisa kit (ml002038; Mlbio, Shanghai, China), according to the manufacturer’s guidance.

**Supplemental Tables and Figures**

**Table S1: List of primers used in the study.**

| Primer | Forward sequence (5’-3’) | Reverse sequence (5’-3’) |
| --- | --- | --- |
| ANF | CTGGGACCCCTCCGATAGAT | TTCGGTACCGGAAGCTGTTG |
| BNP | TTTGGGCTGTAACGCACTGA | CACTTCAAAGGTGGTCCCAGA |
| IL-1β | CTTCCCCAGGGCATGTTAAG | ACCCTGAGCGACCTGTCTTG |
| IL-6 | TTCCATCCAGTTGCCTTCTTG | TTGGGAGTGGTATCCTCTGTGA |
| TNF-α | CCCTCACACTCACAAACCAC | ACAAGGTACAACCCATCGGC |
| Col1 | CCTCAGGGTATTGCTGGACAAC | CAGAAGGACCTTGTTTGCCAGG |
| Col3 | TGACTGTCCCACGTAAGCAC | GAGGGCCATAGCTGAACTGA |
| α-SMA | AGCCATCTTTCATTGGGATGG | CCCCTGACAGGACGTTGTTA |
| β-actin | ACTGCCGCATCCTCTTCCT | TCAACGTCACACTTCATGATGGA |

**Figure S1: Methionine (Met) diet induces a mild HHcy in mice.**

Serum Hcy concentration determined at the end of the experiment, n=5-6 per group. ***: p<0.001.

**Figure S2: FA supplement corrects Met diet-induced HHcy in mice.**

Serum Hcy concentration determined at the end of the experiment, n=5-7 per group. ***: p<0.001.

**Figure S3: Hcy exacerbates AngII-induced hypertrophic response in NRCMs.**

Representative α-actinin fluorescent staining of the Hcy-treated NRCMs and quantitation of NRCM sizes. n=7-8 per group. *: p<0.05; ***: p<0.001.

**Figure S4: FA supplement attenuates HHcy-stimulated hypertrophic response in NRCMs.**

Representative α-actinin fluorescent staining of the FA-treated NRCMs and quantitation of NRCM sizes. n=5-6 per group. **: p<0.01.

**Figure S5: CsA attenuates HHcy-stimulated hypertrophic response in NRCMs.**

Representative α-actinin fluorescent staining of the CsA-treated NRCMs and quantitation of NRCM sizes. n=8-9 per group. ***: p<0.001.
